# The cerebellum: a new key structure in the navigation system

**DOI:** 10.3389/fncir.2013.00035

**Published:** 2013-03-13

**Authors:** Christelle Rochefort, Julie M. Lefort, Laure Rondi-Reig

**Affiliations:** ^1^UPMC Univ Paris 06, UMR 7102Paris, France; ^2^CNRS, UMR 7102Paris, France

**Keywords:** cerebellum, hippocampus, navigation, LTD, self-motion, path integration, place cells, spatial representation

## Abstract

Early investigations of cerebellar function focused on motor learning, in particular on eyeblink conditioning and adaptation of the vestibulo-ocular reflex, and led to the general view that cerebellar long-term depression (LTD) at parallel fiber (PF)–Purkinje cell (PC) synapses is the neural correlate of cerebellar motor learning. Thereafter, while the full complexity of cerebellar plasticities was being unraveled, cerebellar involvement in more cognitive tasks—including spatial navigation—was further investigated. However, cerebellar implication in spatial navigation remains a matter of debate because motor deficits frequently associated with cerebellar damage often prevent the dissociation between its role in spatial cognition from its implication in motor function. Here, we review recent findings from behavioral and electrophysiological analyses of cerebellar mutant mouse models, which show that the cerebellum might participate in the construction of hippocampal spatial representation map (i.e., place cells) and thereby in goal-directed navigation. These recent advances in cerebellar research point toward a model in which computation from the cerebellum could be required for spatial representation and would involve the integration of multi-source self-motion information to: (1) transform the reference frame of vestibular signals and (2) distinguish between self- and externally-generated vestibular signals. We eventually present herein anatomical and functional connectivity data supporting a cerebello-hippocampal interaction. Whilst a direct cerebello-hippocampal projection has been suggested, recent investigations rather favor a multi-synaptic pathway involving posterior parietal and retrosplenial cortices, two regions critically involved in spatial navigation.

## Introduction

Whilst the cerebellum has long been exclusively associated with motor function, its role in cognitive processes has, in the last decades, progressively become apparent. This review will first focus on the original work leading to the major hypothesis that long-term depression (LTD) at parallel fiber (PF)–Purkinje cell (PC) synapses underlies cerebellar motor learning. We then provide an overview of the arguments suggesting that cerebellar processing is also required in cognitive function such as spatial navigation and that it contributes to both hippocampal spatial map formation and optimal goal-directed navigation. The potential computation undertaken by the cerebellum for building hippocampal spatial representation is also discussed. Finally, the possible anatomical pathways involved in this cerebello-hippocampal association are explored.

## Cerebellar LTD and motor learning

LTD refers to an activity-dependent long lasting decrease in synaptic efficacy. This anti-hebbian form of synaptic plasticity was initially discovered in and thought to be unique to the cerebellum (Ito and Kano, 1982; but see Ito, 1989) until it was also described in many other brain areas [e.g., hippocampus (Stanton and Sejnowski, 1989) and cortex (Artola et al., 1990)]. Although Brindley was the first to propose plastic synaptic features to PC (Brindley, 1964), the Marr–Albus theory, which emerged after the fine description of the cerebellar circuitry (Eccles, 1965, 1967), was the one that historically inspired future research. According to this model, the cerebellum acts as a pattern classification device that can form an appropriate output in response to an arbitrary input (Boyden et al., 2004). This implies that the cerebellar circuitry allows adjustments of PF–PC synaptic efficacy, which would enable the storage of stimulus-response associations by linking inputs converging to the cerebellar cortex with appropriate motor outputs. Marr first developed this model by predicting the existence of long-term potentiation (LTP) at PF–PC synapses (Marr, 1969) and Albus modified it two years later by proposing LTD rather than LTP as the learning underlying cellular mechanism (Albus, 1971).

The experimental correlate of the Marr–Albus theory was discovered a few years later by Ito and Kano in 1982. The authors focused on a simple motor learning task and well-defined plastic system: the adaptation of the vestibulo-ocular reflex (VOR). The VOR enables the stabilization of images on the retina during head turns by eliciting eye movements in the opposite direction. Experimental adaptation of this reflex can be obtained by repeatedly displacing the visual stimulus during the head rotation. By studying the VOR circuitry in the rabbit flocculus cerebellar region, Ito and Kano experimentally demonstrated the existence of LTD on PCs after conjunctive stimulation of parallel and climbing fibers (Ito and Kano, 1982; Ito, 1989). Since cerebellar architecture is composed of several uniform modules, it was then suggested that such signal processing may be similar along the entire cerebellum.

Following this work, the implication of LTD in motor learning has been suggested by the observed correlation between altered LTD and impaired motor learning. A series of mouse models lacking LTD has been studied in two main behavioral paradigms, the VOR adaptation and the eyeblink conditioning tasks. In the latter, for which the cerebellum has been shown to be essential (Clark et al., 1984; McCormick and Thompson, 1984a,b), the animal learns to associate a tone (conditioning stimulus) with a corneal air puff (unconditioned stimulus) leading to the eyelid closure. The analysis of mutant mouse models targeting signaling pathways involved in LTD such as the metabotropic glutamate receptor mGluR1 (Aiba et al., 1994), the protein kinase C (PKC) (De Zeeuw et al., 1998; Koekkoek et al., 2003) or the αCaMKII enzyme (Hansel et al., 2006) provided a strong support in favor of the hypothesis that cerebellar LTD is indeed related to cerebellar-dependent motor learning. Nevertheless, a further step to sustain this assertion would be to demonstrate that LTD is effectively induced after cerebellar motor learning.

The current view that cerebellar LTD underlies motor learning was recently challenged as the pharmacological inactivation of cerebellar LTD was not accompanied by a deficit in eyeblink conditioning and in the rotarod test (Welsh et al., 2005). Moreover using a fine behavioral approach designed to selectively eliminate the instructive signal from the climbing fiber (and thus the induction of heterosynaptic LTD) during a VOR adaptation task, it was shown that cerebellar motor learning was completely normal (Ke et al., 2009). In accordance with these findings, the use of three different mutant mouse models targeting specifically late events in the LTD signaling cascade confirmed the dissociation between LTD and simple motor learning tasks (Schonewille et al., 2011).

Interestingly, Burguiere et al. (2010) investigated the role of LTD in an aversive operant conditioning, using a Y-watermaze task in which mice had to learn to associate the correct turn with a stimulus presented before the turn. Inhibition of the PKC crucial for LTD induction did not prevent the animals from learning the stimulus-response “cue–direction” association. In the light of these recent findings, it thus appears that whereas some cerebellar synaptic transmission mechanisms are involved in motor learning, the LTD occurring at PF–PC synapses is not essential. In addition, another form of plasticity, the PF–PC LTP has been proposed to be important for motor learning (Schonewille et al., 2010). Taking into account the different plasticities of the cerebellar cortex including granule cells and PCs network, Gao et al. (2012a) proposed a new conceptual framework called “distributed synergistic plasticity.” They suggest that many forms of synaptic and intrinsic plasticity at different sites combine synergistically to produce optimal output for behavior. This theoretical debate is still ongoing. These mutant mouse models were also an opportunity to extend the study of cerebellar plasticities in other forms of learning abilities, notably in relation to spatial navigation.

## Cerebellum and spatial navigation

**Figure 1:**
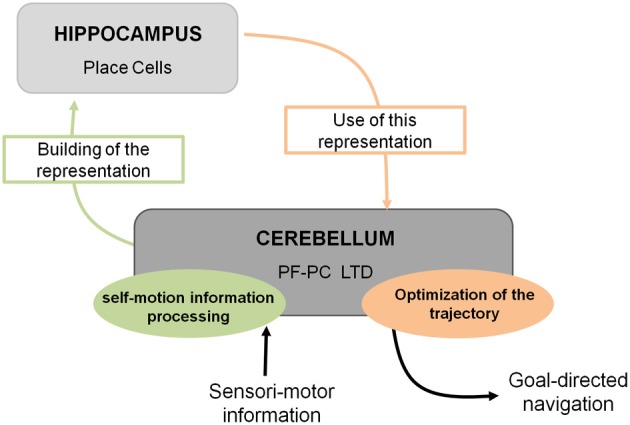
**Cerebello-hippocampal interaction for goal-directed navigation.** The cerebellum contributes to spatial navigation at two levels, first in processing self-motion information to build spatial representation in the hippocampus at the level of place cells, and second in using this spatial representation to perform an optimal trajectory toward a goal. Studying L7-PKCI mice lacking LTD at PF–Purkinje cell synapses, this plasticity has been shown to be involved in both processes.

Spatial navigation is a cognitive function that can be defined as a dual process. Indeed it requires the integration of both self-motion (vestibular, proprioceptive, optic flow, or motor command efferent copy)^1^ and external (visual, olfactory, auditory, or tactile) sensori-motor information to form an internal cognitive representation of the context in which the navigation takes place. This cognitive representation can then be used in order to elaborate an optimal goal-directed path adapted to the context (Figure 1).

Contribution of the cerebellum to cognitive functions such as navigation remains a controversial subject. Indeed, whilst an extensive range of cerebellar functions has been pointed out as early as 1950 (Snider, 1950) and since been completed and corroborated by more recent research, the current understanding of cerebellar functions in cognition suffers from great criticism. For instance, some findings providing important evidence in human that the cerebellum is involved in cognitive function has been refuted based on the general comments that reports of cerebellar activation during cognitive demands are not always replicated and might therefore “be related to actual or planned movements of the eyes, vocal apparatus, or finger” (Glickstein, 2007).

It can however be acknowledged that the view of the cerebellum in cognitive function has evolved with reports describing dysfunction of non-motor processes in patients with cerebellar pathology as well as findings from neuroimaging studies in normal adults (Schmahmann, 1991; Schmahmann and Sherman, 1998; Stoodley and Schmahmann, 2009). For instance, the role of the cerebellum in emotion has been suggested by the difference in the pattern of cerebellar activation induced by distinct types of emotion (Damasio et al., 2000; Baumann and Mattingley, 2012). Implication of the cerebellum in such function has also emerged from a series of investigations using associative fear learning paradigms in patient with cerebellar lesion (see for review Timmann et al., 2010). These results are further supported by studies in rodents, which clearly demonstrated that PF–PC LTP underlies associative memory processes related to fear behavior (for reviews see Sacchetti et al., 2009; Strata et al., 2011). Importantly it has been evidenced that cerebellar LTP was indeed induced by associative fear learning (Sacchetti et al., 2004; Zhu et al., 2007).

The earliest studies combining mental or virtual navigation tasks with brain imaging and focusing on hippocampal and cortical networks reported that cerebellum was also activated during these tasks (Maguire et al., 1998; Ino et al., 2002; Moffat et al., 2006). A few neuroimaging studies using driving simulators showed that a network of brain structures including the cerebellum was specifically activated during driving (Walter et al., 2001; Calhoun et al., 2002; Uchiyama et al., 2003; Horikawa et al., 2005). Findings emerging from patients with cerebellar damage led to diverging conclusions. A series of investigation in children who underwent a resection of cerebellar tumors points toward a role of the cerebellum in visuo-spatial skills (Levisohn et al., 2000; Riva and Giorgi, 2000; Steinlin et al., 2003), although discrepancies exist regarding the part of the cerebellum associated to it. Whereas impaired spatial abilities have been specifically associated to lesions of the left cerebellum in the study of Riva and Giorgi, others works did not find any lateralization (Levisohn et al., 2000). Several studies assessing visuo-spatial abilities in adult cerebellar patient reported that cerebellar lesion leads to an alteration in spatial function (Wallesch and Horn, 1990; Malm et al., 1998; Schmahmann and Sherman, 1998; Molinari et al., 2004), with for some reports a specific involvement of the posterior part of the cerebellum (Schmahmann and Sherman, 1998). However, other reports attribute the observed visuo-spatial deficits of cerebellar patient to unspecific attention impairment rather than spatial neglect (Frank et al., 2007, 2008, 2010). Moreover, in a study assessing the ability of adult subject to navigate without any visual input, patient with cerebellar ataxia displayed trajectories that were even more accurate than control (Paquette et al., 2011), although their angular motion was impaired (Goodworth et al., 2012). Based on the results emerging from both fMRI and cerebellar lesion studies, it has been recently suggested that the cerebellum is part of at least two distinct functional loops, one involved in motor processing and the other involved in cognitive processes (Strick et al., 2009; Ramnani, 2012). Whereas accumulating evidence support the idea that cerebellum participate in both motor and non-motor function, its specific involvement in human spatial navigation remains to be established.

**Figure 2:**
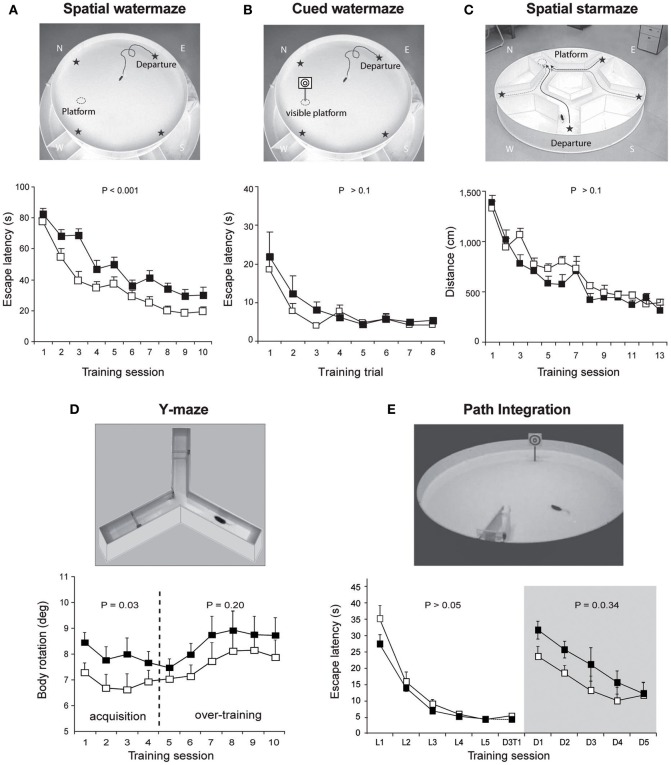
**Overview of the different paradigms used in L7-PKCI mice to assess their spatial navigation performances. (A)** The spatial version of the Morris watermaze, in which a hidden platform can be found using the configuration of distal visual cues around the maze revealed spatial learning deficits in L7-PKCI mice. **(B)** These lower performances are not due to a deficit in visual guidance abilities, as revealed by subsequent training in the cued version of this maze. In this task, L7-PKCI mice correctly found the platform location indicated by a proximal visual cue. **(C)** The absence of deficit in the starmaze, in which alleys help to execute efficient trajectories toward an invisible platform, suggests that while L7-PKCI can learn to locate a goal using distal visual cues, they are unable to execute a direct trajectory toward it. **(D)** The use of a single intersection maze called the Y-maze enables analysis of trajectory quality. Trajectories optimization was quantified by the body rotation parameter which measures the angle between 3 successive points of the trajectory and is averaged over the whole path. Body rotation was specifically impaired in L7-PKCI mice during the acquisition of the task. **(E)** The inability of L7-PKCI mice to navigate in the dark (i.e., using self-motion information) was revealed in a path integration task, in which mice have to find a platform in a constant location and from a fixed departure point with an alley guiding the initial orientation of the body. In this task, mice first learn the path in the light and then reproduce it in the dark. **(A,B,C)** Data from Burguiere et al. (2005); **(D)** data from Burguiere et al. (2010); **(E)** data from Rochefort et al. (2011). N, W, E, S: North, West, East, and South, respectively. Stars on the pictures in **(A,B,C)** indicate each possible departure point.

In non-human primates, one of the first reports on the contribution of the cerebellum to spatial learning abilities emerged in the 80's. This study carried out on adult monkeys with experimental lesions of the deep cerebellar dentate nucleus revealed an impaired performance in the spatial parameter of a visuo-motor task involving a goal-directed movement of the arm (Trouche et al., 1979). These results represented a first step toward an enlarged view of cerebellar functions, encompassing more complex spatial learning task. The role of the cerebellum in spatial learning has also been investigated using water maze tasks in rodents given the reduced impact cerebellar lesions exert on swimming movements (see review in Lalonde and Strazielle, 2003). However, whilst several authors emphasized the navigation deficit in cerebellar mutant models, a recurrent problem has been to dissociate between the navigation process deficit *per se* and motor-related problems. Therefore, rodents were tested in cued or spatial learning paradigms of a water maze in order to evaluate their visuo-motor abilities or their spatial navigation abilities respectively (see Figure 2 for more details about the paradigms). Several cerebellar mutant mice such as Grid2^Lc^, Rorα^sg^, reeler and weaver presented deficits in both cued and spatial learning (see review in Rondi-Reig and Burguiere, 2005). However, these natural mutations were relatively large and affected the whole cerebellar organization. Nevertheless, another cerebellar mutant mouse (Nna1^pcd^) which displays a postnatal specific degeneration of virtually all cerebellar PCs (Mullen et al., 1976) was able to perform the cued but not the spatial version of the task indicating that the severe spatial navigation deficit of this mutant was not simply due to motor dysfunction (Goodlett et al., 1992). Similarly, hemicerebellar lesions led to deficits in both spatial and cue version of the MWM (Petrosini et al., 1996), whereas more restricted lesions to the lateral cerebellar cortex, the dentate nucleus (Joyal et al., 2001; Colombel et al., 2004) or the Purkine cell layer (Gandhi et al., 2000) reveals a specific impairment in the spatial version of this task. Altogether, based on the specificity of the behavioral and neurobiological alterations, these data clearly supported the hypothesis that the cerebellum is involved in spatial learning (see reviews in Petrosini et al., 1998; Molinari and Leggio, 2007).

The accumulation of evidence supporting a role of the cerebellum in navigation raised the question of the potential roles of the two major cerebellar inputs, the olivo-cerebellar input (climbing fiber) and the mossy fiber–granule cells–PF input. Rondi-Reig et al. (2002) tested rats with lesion of climbing (CF) and/or PF inputs of the cerebellum in either the cued or the place protocol of the water maze. Rats with a lesion of CF associated with partial or total lesion of PF presented a deficit in the latency to find the platform in the spatial version of the task but not in the cued one. Interestingly a difference appeared between the CF and PF lesion in the initial body orientation relative to the platform. Animals presenting a lesion of the PF were unable to learn how to orient their body toward the non-visible platform and opted instead for a circling behavior, whereas animals with lesion of the CF were still able to reach control level. These results indicated a substantial role of the PF cerebellar inputs in navigation (Rondi-Reig et al., 2002) and pointed toward an underlying mechanism occurring at the PC synapse.

Recent use of the L7-PKCI transgenic model, in which the PKC dependent LTD that occurs at PF–PC synapses is altered, brought new insight regarding the process performed by the cerebellum (Burguiere et al., 2005, 2010; Rochefort et al., 2011) (Figure 1). Using this L7-PKCI model in an operant conditioning task, our team highlighted the idea that cerebellar LTD is not required for the learning of a stimulus-response association but is rather involved in the optimization of a motor response during a goal-directed navigation conditioning task (Burguiere et al., 2005, 2010). Using a behavioral protocol assessing specifically path integration of the L7-PKCI mice (i.e., the ability to navigate using self-motion information only), we revealed an implication of cerebellar LTD in the formation of the self-motion based internal spatial map encoded in the hippocampus. Indeed, mice lacking this form of cerebellar plasticity presented impaired hippocampal place cell firing properties. Interestingly, the deficit in the hippocampal place code was observed only when mice had to rely on self-motion information. Subsequently, mice were tested in a path integration task, in which they had to find a platform in a constant location and from a fixed departure point with an alley guiding the initial orientation of the body (Figure 2). Mice first learned the path in the light and then had to reproduce it in the dark. Consistently with their hippocampal place cell alteration, L7-PKCI mice were unable to navigate efficiently toward a goal in the absence of external information (Figure 2). Princeps studies on navigation in rats suggested that the cerebellum is not required for the retention of a learned path in a maze habit task with guiding alleys, even in the absence of vision (Lashley and McCarthy, 1926). It is possible that the fact that mice are overtrained and the presence of alleys guiding the animal movement had hidden a potential deficit. Likewise, L7-PKCI mice were also not deficient in the starmaze, a navigation task in which mice swim only within alleys (Burguiere et al., 2005).

The deficit in the spatial map observed in L7-PKCI mouse model brought the first evidence of a functional interaction between the cerebellum and the hippocampus in the acquisition of a spatial representation required to perform path integration (Rochefort et al., 2011). According to these findings, cerebellar LTD might participate in the mental construction of the representation of space whose seat is in the hippocampus, suggesting that the cerebellum takes part in the representation of the body in space. The next section is focused on describing the mechanisms by which the cerebellum might participate in navigation by processing and combining multimodal self-motion information and give pertinent information about body location in space.

## Cerebellar contribution to navigation information processing

**Figure 3:**
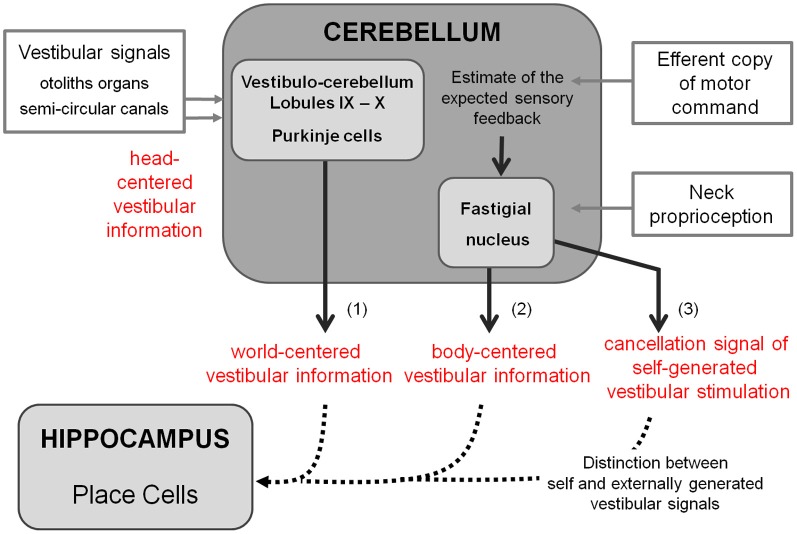
**Detailed cerebellar processing of self-motion information that can be used for building spatial representation.** This figure represents the ascendant branch of Figure 1 and highlights the cerebellar contribution to building spatial representation. Based on the existing literature, cerebellar processing of self-motion information could involve three different computations: **(1)** The combination of otolith and semi-circular signals to convert head centered vestibular information into world centered vestibular information. **(2)** The integration of neck proprioceptive information with head motion vestibular information to compute an estimate of body motion in space. **(3)** The hypothesis proposed by Cullen et al. (2011) of a possible production of a cancellation signal to suppress self-generated vestibular stimulation due to active movements. This computation implies using the efferent copy of motor command to predict expected sensory feedback and to compare it to the effective proprioceptive signal (Roy and Cullen, 2004). Such a cancellation allows distinguishing between self-and externally-generated vestibular signals. These transformations are required to provide the hippocampus with the appropriate self-motion information (dotted lines).

As previously explained, spatial navigation is an active process that requires the accurate and dynamic representation of our location, which is given by the combination of both external and self-motion cues. Vestibular information has been shown to be crucial for spatial representation (Stackman et al., 2002), spatial navigation (Stackman and Herbert, 2002; Smith et al., 2005), and specifically path integration (Wallace et al., 2002). However, vestibular information by itself does not provide sufficient information to properly locate in an environment. Coherent body motion information is indeed given by the combination of multiple sources of idiothetic information including vestibular, proprioceptive, optic flow, and motor command efferent copy signals. Figure 3 suggests the role of the cerebellum in such integration.

**Figure 4:**
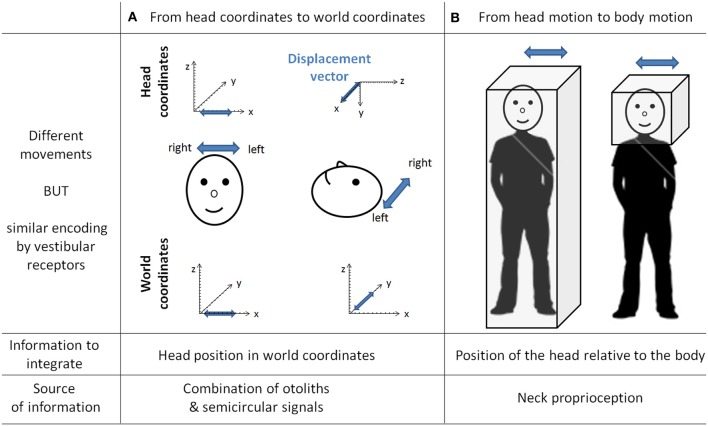
**The need for transformation of the vestibular signals.** As the vestibular organs are located in the head, vestibular signal is detected in head coordinates. This implies several transformations of the vestibular signal to correctly compute body motion in space. This figure gives two examples of different movements similarly encoded by vestibular receptors. In column **(A)** is a linear displacement from left to right, with the head either vertical or horizontal. Indeed both movements are identical in the head reference frame [displacement vectors (in blue) project onto the x-axis] whereas they are different in the world coordinates (displacement vectors project either onto the x-axis or onto the y-axis). These two movements can be distinguished by taking into account the head position in space, which can be extracted from the combination of semicircular and otolith organs signals (Yakusheva et al., 2007). Column **(B)** illustrates two movements corresponding to the same head motion in space, but different body motions in space (i.e., on the right the body is stationary). These two movements can be distinguished by integrating information about the position of the head relative to the body (that is, the neck curvature, given by neck proprioceptors).

Vestibular information is first detected in the inner ear by the otoliths organs for the linear component and by the semi-circular canals for the rotational component. As receptor cells are fixed to the head bone, vestibular signals are detected in a head reference frame (Figure 4). This means for example that based on semicircular signals only, a rotation of the head upright relative to the vertical axis cannot be distinguished from a rotation of the head horizontal relative to the horizontal axis. In other words, semicircular canal information alone does not discriminate vertical or horizontal body position. To compute the movement of the body in space, vestibular information needs to be integrated relative both to the body (taking into account the relative position of the head and the body, given by the neck curvature) and to the world, converting the signal initially in head-fixed coordinates into a signal in world-frame coordinates (taking into account gravity). These computations are not necessarily successive and result from the integration of different types of signals. Several recent studies showed that these two reference frame transformations occur in different cerebellar subregions. An elegant report recently pointed out that the cerebellar cortex computes the head-to-world reference frame conversion by combining semicircular and otolith organs inputs (Yakusheva et al., 2007). This computation takes place in the lobules 9 and 10 of the cerebellum and involves GABA transmission (Angelaki et al., 2010).

Head-to-body frame transformation seems to occur in the cerebellar fastigial nucleus. This region contains indeed a subpopulation of neurons (50%)—one synapse downstream the PCs—that has been shown to encode motion in body coordinates (Kleine et al., 2004; Shaikh et al., 2004). More recently, this idea has been further supported by the demonstration that fastigial neurons respond to both vestibular and neck proprioception, and specifically encode body movement in space (Brooks and Cullen, 2009). However, since head to body position has also been shown to modulate PC activity in the cerebellar anterior vermis in decerebrate cats (Manzoni et al., 1999), meaning that PCs also receive neck proprioceptive information, one cannot exclude that the head to body frame transformation might also take place in the cerebellar cortex.

Another implication of the cerebellum in the sensory processing involved in spatial navigation has been highlighted by studies on the cancellation of self-generated vestibular signals. During spatial navigation, displacement of the body in the environment undoubtedly generates stimulations of vestibular receptors. This includes translational stimulations corresponding to the displacement vector as well as rotational stimulations due to head and body reorientation. However, vestibular stimulations are not perceived, meaning that these self-generated signals have been canceled out, enabling reliable detection of stimuli from external sources. Crucial to navigation, the ability to distinguish self-generated vestibular signals coming from an active movement allows proper integration with other types of idiothetic signals to produce an accurate estimate of body movement, which forms the basic computation for path integration.

A particular population of neurons within vestibular nuclei termed Vestibular Only (VO) are selectively active during passively applied movements (McCrea et al., 1999; Roy and Cullen, 2001). The lack of response during active movements implies that self-generated vestibular signals are indeed canceled. Such cancellation requires knowledge about the currently performed movement provided by the combination of the different self-motion signals, and in particular the efferent copy of the motor command and proprioception. Because the VO neurons are modulated by neither proprioceptive inputs nor efferent copy of motor command when presented in isolation to alert animals, some authors suggested that a cancellation signal arrives from higher structures in the case of active movements (Roy and Cullen, 2003, 2004). Moreover, Roy and Cullen (2004) showed that during active movements, this cancellation signal occurs only if the actual movement matches the intended one. These authors proposed that, using the efferent copy of motor command, an internal model of proprioception is computed and compared to the actual proprioceptive signal. If it matches, a cancellation signal is generated and sent to the vestibular nuclei. The exact location of the cancellation signal generation remains to be determined. Such a region should receive proprioceptive signals, efferent copies of the motor commands or an estimate of the expected sensory consequences of actions, and vestibular signals. For these reasons Cullen et al. (2011) proposed that the cerebellar rostral fastigial nucleus would be a good candidate. Indeed it does receive inputs from the cerebellar cortex—whose function is thought to be (among others) the generation of sensory prediction—neck proprioception from the central cervical nucleus and the external cuneate nucleus and vestibular inputs from the vestibular nucleus (Voogd et al., 1996). Additionally, recordings in fastigial nucleus (Brooks and Cullen, 2009) strongly suggested that the integration of proprioceptive and vestibular information takes place in the rostral fastigial nuclei during passive movement. Whether this integration occurs during active movement and is used to generate a cancellation signal remains to be demonstrated.

Thus, the cerebellum is likely to act in a heterogeneous manner, involving several subregions in the cerebellar cortex and deep nuclei for the transformation of the reference frame adapted to navigation in space and for the cancellation of self-generated vestibular signals, enabling a focus on pertinent external stimulation for optimal path. The information, adequately transformed, is subsequently conveyed to the hippocampus (Figure 3).

The exact network and plasticities involved in this computation during navigation remains to be elucidated. Deficits observed in the L7-PKCI mice suggest that cerebellar PF–PCs LTD is involved in such computation and plays an important role in self-motion based hippocampal space representation.

## Anatomical and functional relation between cerebellum and forebrain navigation areas

Demonstration that the cerebellum assists navigation at least in part by participating in the building of the hippocampal spatial map (Rochefort et al., 2011) implies that these structures are interconnected. Therefore, the cerebellum communicates either directly with the hippocampal system or with the forebrain navigation areas connected to it. Interestingly, a functional interaction between the hippocampus and the cerebellum has recently been supported by two studies conducted in rabbits using the hippocampal-dependent *trace* version of the eyeblink conditioning task (Hoffmann and Berry, 2009; Wikgren et al., 2010). Both investigations clearly demonstrate that during trace eyeblink conditioning, theta oscillation (3–7 Hz) occurs in the lobule HVI and the interpositus nucleus of the cerebellum and is synchronized with hippocampal theta oscillation. The cerebellar theta oscillations appeared to depend on the hippocampal theta rhythm. These data demonstrate that the hippocampus and the hemispheric lobule HVI of the cerebellum, which is involved in the stimulus-response association of the trace eyeblink conditioning, can synchronize their activity during specific cognitive demands. Whilst the data from Hoffmann and Berry (2009) suggest that this coordination enhances the associative learning abilities, Wikgren et al. (2010) did not observe a link between hippocampo-cerebellar synchronization and learning performances. Regardless, the latter investigations invite speculation on the possibility of multiple synchronization areas between the hippocampus and the cerebellum, which may be required for spatial navigation.

One important question raised by these findings is the anatomical circuitry underlying such functional interaction. Some evidence suggests a direct anatomical link between the hippocampus and the cerebellum. In cat and monkey, fastigial nucleus stimulation consistently evoked responses bilaterally in the rostro-caudal region of the hippocampus at delays indicating a monosynaptic connection (Heath and Harper, 1974; Snider and Maiti, 1976; Heath et al., 1978; Newman and Reza, 1979). Heath and Harper (1974) also found degenerated fibers in the hippocampus following lesion of the fastigial nucleus, meaning that these fibers could directly originate from the deep cerebellar nucleus. Hippocampal responses following posterior vermis stimulation were also reported (Heath et al., 1978; Newman and Reza, 1979) but not after stimulation of other cerebellar subregions. However, these observations have not, so far, been confirmed by anatomical investigations, possibly because of the potentially low number of implicated fibers.

Nevertheless, a recent study combining retrograde tracing and degeneration analysis after hippocampal lesion demonstrated a direct projection from the hippocampal formation to the cerebellum in chicken (folia VI–VIII) (Liu et al., 2012). The existence of a hippocampo-cerebellar projection does not imply a backward projection from the cerebellum to the hippocampus, which could explain the influence of cerebellar plasticity in shaping hippocampal place cell properties (Rochefort et al., 2011). However, tracing studies performed in the monkey in the last decade suggest a general organizational principle of the cerebello-cortical system where different areas of the neocortex are reciprocally connected to the cerebellum in closed loops (Clower et al., 2001; Middleton and Strick, 2001; Kelly and Strick, 2003; Prevosto et al., 2010). A direct cerebello-hippocampal projection remains to be discovered.

Alternatively, the cerebellum could interact with the hippocampus through multi-synaptic connections via the forebrain navigation circuit. Evidence from rat studies suggests that this interaction may take place via multiple pathways. The cerebellum reaches the forebrain mainly through the projection from the deep cerebellar nuclei toward the thalamus. Interestingly, substantial cerebellar inputs are found in the central-lateral thalamic nucleus (Haroian et al., 1981; Angaut et al., 1985; Aumann et al., 1994). The central lateral nucleus projects to both the posterior parietal and the retrosplenial cortices (Van der Werf et al., 2002), two cortical areas particularly involved in spatial navigation.

The posterior parietal cortex (PPC) is a multi-modal cortical area integrating self-motion and visuo-spatial information (Snyder et al., 1998; Save and Poucet, 2009). Its role in spatial navigation has been recently enlightened by the discovery of rat PPC cells the activity of which is tuned to self-motion and acceleration irrespective to the animal location or heading (Whitlock et al., 2012). The presence of cells encoding movement in an egocentric reference frame thus makes the PCC a primary candidate for the reception of cerebellar information and its transmission to other navigation areas. Such hypothesis is further supported by the close interaction between PPC and cerebellar lobule VIIa and Crus I and II showed in a human resting state functional connectivity study (O'Reilly et al., 2010). Moreover, combining anterograde and retrograde tracing, studies in both rat and primate confirmed that the PPC receives cerebellar input from the interposed and lateral nuclei via a thalamic relay in the central-lateral and ventro-lateral nuclei (Amino et al., 2001; Clower et al., 2001; Giannetti and Molinari, 2002; Prevosto et al., 2010). The existence of reciprocal connections from the parietal cortex to the cerebellum has not been documented so far in the rodent but cerebello-parietal interaction could follow the closed-loop architecture of cerebro-cerebellar interactions. Moreover in monkey, the homologous area to the rat PPC (the area 7) has indeed been shown to project to the cerebellar hemispheres via the pontine nucleus (Glickstein et al., 1985; Dum et al., 2002). Such projection could contribute substantially to multisensory integration (Glickstein, 2003).

The retrosplenial cortex is also thought to be involved in the allocentric-to-egocentric transformation process (Vann et al., 2009). Indeed, retrosplenial inactivation has been shown to impair allocentric navigation and path integration as well as field location of hippocampal place cells (Cooper et al., 2001; Cooper and Mizumori, 2001; Whishaw et al., 2001). This cortical area also contains head direction cells which are found in a network of structures (Taube, 2007) and were recently shown to underlie a rodent's sense of direction during path integration (Valerio and Taube, 2012). Head direction signal is prominently dependent on vestibular information (Stackman et al., 2002) and is believed to be generated subcortically and then processed by higher structures such as the retrosplenial cortex (Taube, 2007).

**Figure 5:**
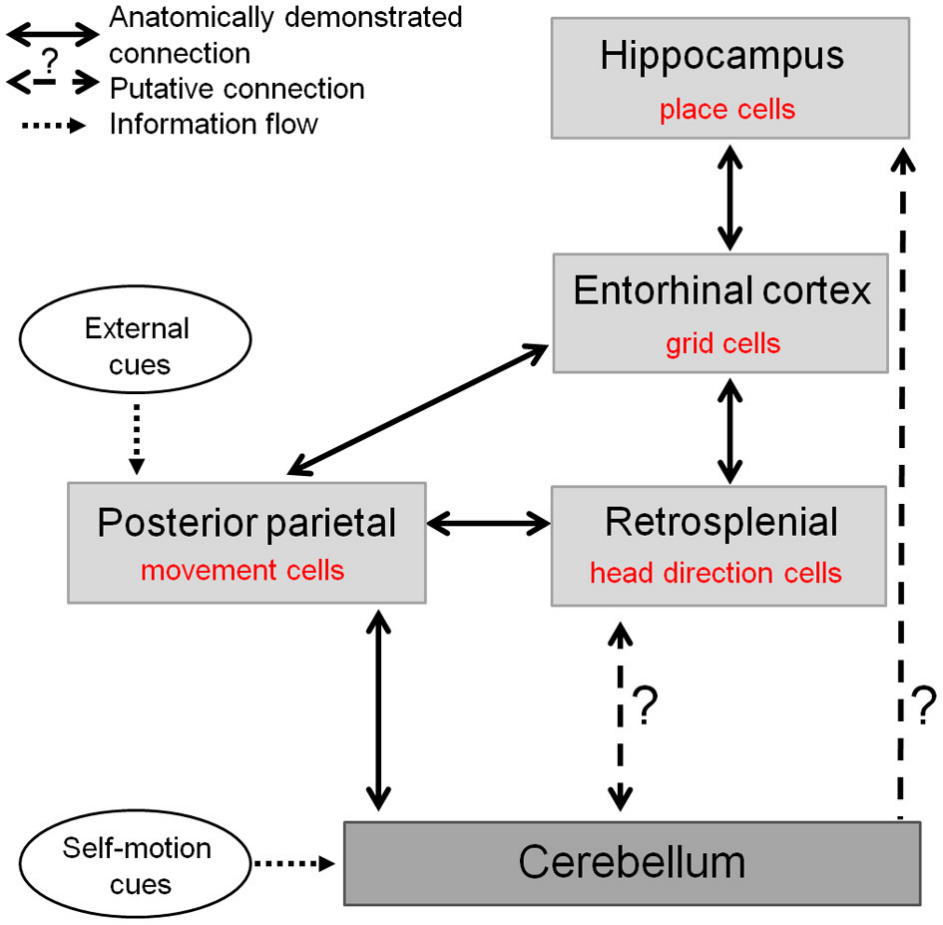
**The cerebellum in the anatomo-functional circuit underlying spatial navigation.** Navigation system comprises a whole network of structures: (1) the hippocampus containing «place cells», which correlate with animal's location in space and underlie animal's spatial representation (O'Keefe and Dostrovsky, 1971), (2) the medial enthorinal cortex, containing «grid cells» which fire according to a grid-like pattern and are thought to constitute the metric system of the brain (Hafting et al., 2005), (3) a network of structures—among which the retrospenial cortex—containing «head direction cells», specific for a given direction of the head in space (Taube et al., 1990), and (4) posterior parietal cortex containing «movement cells», encoding self-motion and acceleration (Whitlock et al., 2012). The cerebellum takes part in this navigation system as it shapes hippocampal place cells properties (Rochefort et al., 2011). This contribution could occur either through a direct projection to the hippocampus or via a multi-synaptic connection involving a thalamic relay, to the posterior parietal cortex or the retrosplenial cortex.

Therefore, the cerebellum may contribute to two major circuits crucial for the representation of space in the hippocampal system (Figure 5): one comprising the retrospenial cortex more closely associated to the vestibulo-cerebellum, and the other involving the PPC receiving inputs from deep cerebellar nuclei and possibly involved in planning and execution of navigation behavior. Nevertheless the precise anatomical pathways between cerebellum and hippocampus activated during spatial navigation remain to be elucidated.

## Concluding remarks

Recent converging evidence demonstrates the importance of the cerebellum in spatial navigation. Such implication in the navigation system at the hippocampal level or in forebrain navigation areas has been elucidated using electrophysiological, anatomical, and behavioral analyses in both human and animal models. Although the cerebellar network does not encode a spatial map of the environment, it does participate in map formation in the forebrain navigation areas by specifically encoding and computing self-motion information from different sources required to build the representation of the body in space. PF–PC LTD is implicated in this process, and other as yet to be determined modes of cerebellar plasticity may participate as well. The recent development of tetrodes multi-unit recordings in rat's cerebellum (de Solages et al., 2008; Gao et al., 2012b) opens new perspectives to unravel the cerebellar computation occurring during goal-directed navigation. In order to unravel the precise contributions of the cerebellum to the processing of information during navigation, such technique will have to be combined with the use of mutant animals bearing specific cerebellar plasticity deficits.

### Conflict of interest statement

The authors declare that the research was conducted in the absence of any commercial or financial relationships that could be construed as a potential conflict of interest.

## Footnotes

^1^See Glossary.

## Glossary

Vestibular organs: Semicircular canals and otolith organs. The latter detect linear acceleration whereas the former are sensitive to angular acceleration.
Proprioception: Perception of the relative position of the different parts of one's body using information from proprioceptors (i.e., stretch receptors located in the muscles, tendons, and joints).
Efferent copy of motor command: It has been suggested that during an active movement, while the motor cortex sends a command to the periphery, a copy of the motor command is also generated and could be used to generate a prediction of the sensory consequences of the intended movement.
Optic flow: The displacement of images on the retina due to the relative motion between the observer and the scene. The displacement speed can be used to estimate one's proper acceleration.
